# Religious affiliation and perceptions of healthcare access during and after COVID-19 in Poland

**DOI:** 10.3389/fpubh.2025.1520575

**Published:** 2025-03-17

**Authors:** Magdalena Tuczyńska, Maja Matthews-Kozanecka, Ewa Baum

**Affiliations:** ^1^Department of Social Sciences and the Humanities, Poznan University of Medical Sciences, Poznan, Poland; ^2^Division of Philosophy of Medicine and Bioethics, Poznan University of Medical Sciences, Poznan, Poland

**Keywords:** COVID-19, faith, religiosity, healthcare, pandemic

## Abstract

**Introduction:**

In response to the unprecedented impact of the COVID-19 pandemic on healthcare systems and social life worldwide, this study examines the role of religion in shaping perceptions of healthcare access in Poland during and after the pandemic.

**Methods:**

The research is based on anonymous surveys conducted among adult Poles during the third wave of the pandemic and in the post-pandemic period, with participants divided by the timing of their responses. The research employed a cross-sectional survey design with a validated questionnaire. Data collection occurred during the third wave of the pandemic and the post-pandemic period. The questionnaire incorporated demographic questions and assessed the significance of religion in respondents' lives using a Visual Analogue Scale (VAS) for healthcare accessibility.

**Results:**

Participants (*n* = 541) were recruited through online and paper-based surveys, meeting the inclusion criteria of being aged 18 or older and residing in Poland. Findings indicate that Roman Catholic respondents rated healthcare accessibility higher than non-religious individuals, potentially due to social and community support. However, statistical analysis revealed no significant differences in actual healthcare access among groups, suggesting systemic factors played a larger role.

**Discussion:**

These results highlight religion as a source of emotional support rather than a determinant of healthcare access.

## Introduction

The COVID-19 pandemic, caused by the new SARS-CoV-2 coronavirus, has become one of the biggest health challenges of the 21st century. Since its outbreak in late 2019 in the Chinese city of Wuhan, the virus has spread rapidly around the world, turning into a global health threat (1). The World Health Organization (WHO) declared a pandemic state in March 2020, initiating a global effort to curb the spread of the virus (2). The pandemic has had a major impact on healthcare systems, which have had to quickly adapt to the unprecedented scale of the disease. Hospitals and medical facilities around the world struggled with shortages of protective equipment, ventilators and ICU space. Many countries instituted emergency measures such as lockdowns, border closures, quarantines and orders to wear masks to slow the spread of the virus and ease the burden on health systems. These measures, while necessary, have had far-reaching social and economic consequences (3). The pandemic also revealed deep inequalities in access to healthcare, both between countries and within individual societies. Lower-income countries had greater difficulty accessing vaccines, modern therapies and medical equipment. In societies affected by the pandemic, the older adults, ethnic minorities and low-income people were more vulnerable to the severe health and economic impacts of COVID-19 (4, 5). On 5 May 2023, the WHO declared that COVID-19 is no longer a public health emergency of international concern (PHEIC). However, this does not mean that the virus no longer poses a threat to global public health. The virus continues to affect people around the world, and there is always a chance that new variants will emerge to cause new cases and fatalities. Following the announcement, however, the WHO has shifted its focus from emergency response to managing COVID-19 along with other infectious diseases (6, 7).

Access to healthcare is a complex, multidimensional concept that includes physical availability, financial affordability, and sociocultural acceptability of medical services (8, 9). While traditional models of healthcare access focus on geographic and economic barriers, more recent research highlights the role of social and psychological factors, including trust in healthcare institutions and willingness to seek care (10). Religious affiliation significantly shapes perceived access to healthcare by influencing health-seeking behaviors, adherence to medical recommendations, and attitudes toward treatments such as vaccination (11, 12). Studies suggest that faith-based communities provide crucial social support that can improve access to healthcare resources, yet at times, religious beliefs may also act as a barrier to seeking medical help (13, 14).

Poland's healthcare system is based on universal healthcare coverage, with the National Health Fund (NFZ) providing access to medical services for all insured citizens. However, regional disparities, long waiting times, and limited access to specialists have historically posed challenges, which were exacerbated by the COVID-19 pandemic (15). During the pandemic, public hospitals were overwhelmed, leading to increased reliance on private healthcare services and faith-based charitable organizations to fill critical gaps (16). Religious institutions play a significant role in Polish society, with over 90% of the population identifying as Christian, and Roman Catholicism being the predominant faith (17). The Catholic Church has traditionally been influential in public discourse, including matters of healthcare ethics, medical policy, and social assistance. During the COVID-19 pandemic, religious organizations facilitated mental health support, social services, and communication regarding vaccination policies, particularly in rural areas where healthcare resources were scarce (18). Despite this influence, the extent to which religious affiliation impacted individuals' perceptions of healthcare access during the pandemic in Poland remains largely unexplored.

Religion and health are two important aspects of human life that intertwine and influence each other in various ways. For centuries, people have looked to religion for support and understanding of their health problems, believing that there is a deeper meaning and purpose to life that can help overcome physical and mental difficulties (19). Modern scientific research increasingly confirms that religiosity can have a significant impact on an individual's health, influencing his or her wellbeing, treatment processes and approach to medical care (20). The COVID-19 pandemic that erupted at the end of 2019 has created unique circumstances for analyzing the relationship between religiosity and health. The global health disruption caused by the new SARS-CoV-2 coronavirus has had a profound impact on the lives of billions of people around the world, causing not only physical, but also emotional and spiritual challenges. Faced with uncertainty, fear of illness and death, and social isolation, many people have turned to religion as a source of comfort and strength. Religiosity was found to be an important factor in how people responded to the pandemic and coped with its consequences. Studies show that people who were more religious often exhibited greater psychological resilience, better coping mechanisms to deal with stress and a higher quality of life in the face of adversity (21). At the same time, religious beliefs influenced attitudes toward health and medical care, shaping decisions about treatment, vaccination and adherence to preventive measures (22).

In numerous places around the world, the pandemic caused changes in religious practices and religious approaches. In some cases, due to pandemic-related restrictions, people were unable to access traditional worship sites, which may have dampened the intensity of religious practices (23). In other cases, the pandemic may have prompted people to practice more individual forms of religious practices, due to social isolation and restrictions on public gatherings, including religious gatherings (services, prayers, or pilgrimages) (24). The influence of religion on healthcare access and pandemic response varied significantly across different regions. For example, in the United States, some religious communities resisted public health measures, including mask mandates and vaccinations, citing religious freedom concerns (25). Conversely, in South Korea, religious gatherings were identified as super-spreader events, leading to stricter regulations on places of worship and public backlash against religious groups (26). In the Middle East, religious leaders in countries like Saudi Arabia and Iran issued fatwas encouraging vaccination and supported government-led pandemic measures (27). In Brazil, evangelical churches significantly influenced public discourse, with some pastors spreading skepticism toward vaccines, while others actively supported community-based healthcare initiatives (28). These examples illustrate how religious institutions and beliefs shaped both compliance with and resistance to public health measures, demonstrating that the role of religion in healthcare access during the pandemic was highly contextual.

The COVID-19 pandemic has significantly impacted global healthcare systems, exposing deep inequalities in access to medical care. Poland, like other nations, faced shortages in medical resources and implemented emergency measures such as lockdowns and telemedicine services. However, beyond structural challenges, individuals' religious beliefs influenced their perceptions of healthcare accessibility. Previous studies suggest that religion provides emotional support during crises and may influence healthcare-seeking behaviors (20, 23). In Poland, where Roman Catholicism is predominant, religious institutions played a crucial role in supporting healthcare access through community aid programs. Patients' religiosity and forms of expression can influence various aspects of their behavior, including their utilization of healthcare services and perceptions of accessibility to healthcare services. However, the extent to which religion affected perceived access to healthcare during the pandemic remains underexplored. This study aims to fill this gap by comparing perceptions of healthcare accessibility among individuals of varying religious affiliations before, during, and after the pandemic.

## Materials and methods

This cross-sectional study was conducted using an anonymous, self-administered questionnaire, which was approved by the Bioethics Committee at Poznan University of Medical Sciences (reference number 484/21) and complied with the guidelines of the Helsinki Declaration. The research tool used to assess patients' perceptions of healthcare access in Poland before and during the COVID-19 pandemic was a custom-designed questionnaire developed by the authors in Polish. The survey was conducted among residents of Poland, with data collection occurring during two distinct time periods: the final period of the third wave of the COVID-19 pandemic (July 8–August 11, 2021), referred to as the “pandemic group,” and the post-pandemic period (December 8, 2023–January 11, 2025), referred to as the “post-pandemic group.” Due to the anonymous nature of the questionnaire, the same individuals who participated in the first phase could not be re-surveyed in the post-pandemic phase. As a result, two independent groups of participants were surveyed. In both cases, the same inclusion criteria were applied: respondents had to be aged 18 or older, reside in Poland, and provide voluntary informed consent to participate in the study.

The questionnaire used for both groups of respondents was identical and consisted of two sections. The first section included demographic questions related to gender, religious affiliation, and the significance of religion in daily life, among others. The second section assessed respondents' perceptions of healthcare accessibility in Poland before and during the COVID-19 pandemic. To evaluate perceived accessibility, the questionnaire utilized a Visual Analog Scale (VAS), where “0” represented “very poor” access and “10” indicated “very good” access. The reliability of the questionnaire was validated using Cronbach's α coefficient (α = 0.85), confirming its internal consistency. The instrument was adapted to the Polish healthcare context and underwent validation through expert assessment and internal consistency analysis.

As this study focuses on the perceived accessibility of healthcare services in relation to respondents' religiosity, only the most relevant, identical questions were selected from the questionnaire. This selection was necessary because the original questionnaire covered a broader range of topics. The selection process was based on the authors' evaluation, expert opinions from public health specialists, and existing literature on healthcare access and the influence of religion on health behaviors. The questionnaires were distributed in both paper and digital formats. Paper-based surveys were administered in the clinical departments of the Poznan University of Medical Sciences, while the online version was disseminated through Google Forms. Recruitment included hospital patients and members of the general community, ensuring a diverse and representative sample.

The collected data were subjected to statistical analysis. Statistical calculations were conducted using Statistica 13 (TIBCO Software Inc.) and PQStat (PQStat Software). The significance level was set at α = 0.05, and results were considered statistically significant when *p* < α. To compare variables, the Kruskal-Wallis test was used. The χ^2^ test of independence or the Fisher-Freeman-Halton test was applied to examine correlations. To determine whether changes in scores were statistically significant, the Wilcoxon test was employed. Lastly, to evaluate relationships between categorical variables, the χ^2^ test of independence was conducted.

## Results

**Figure 1:**
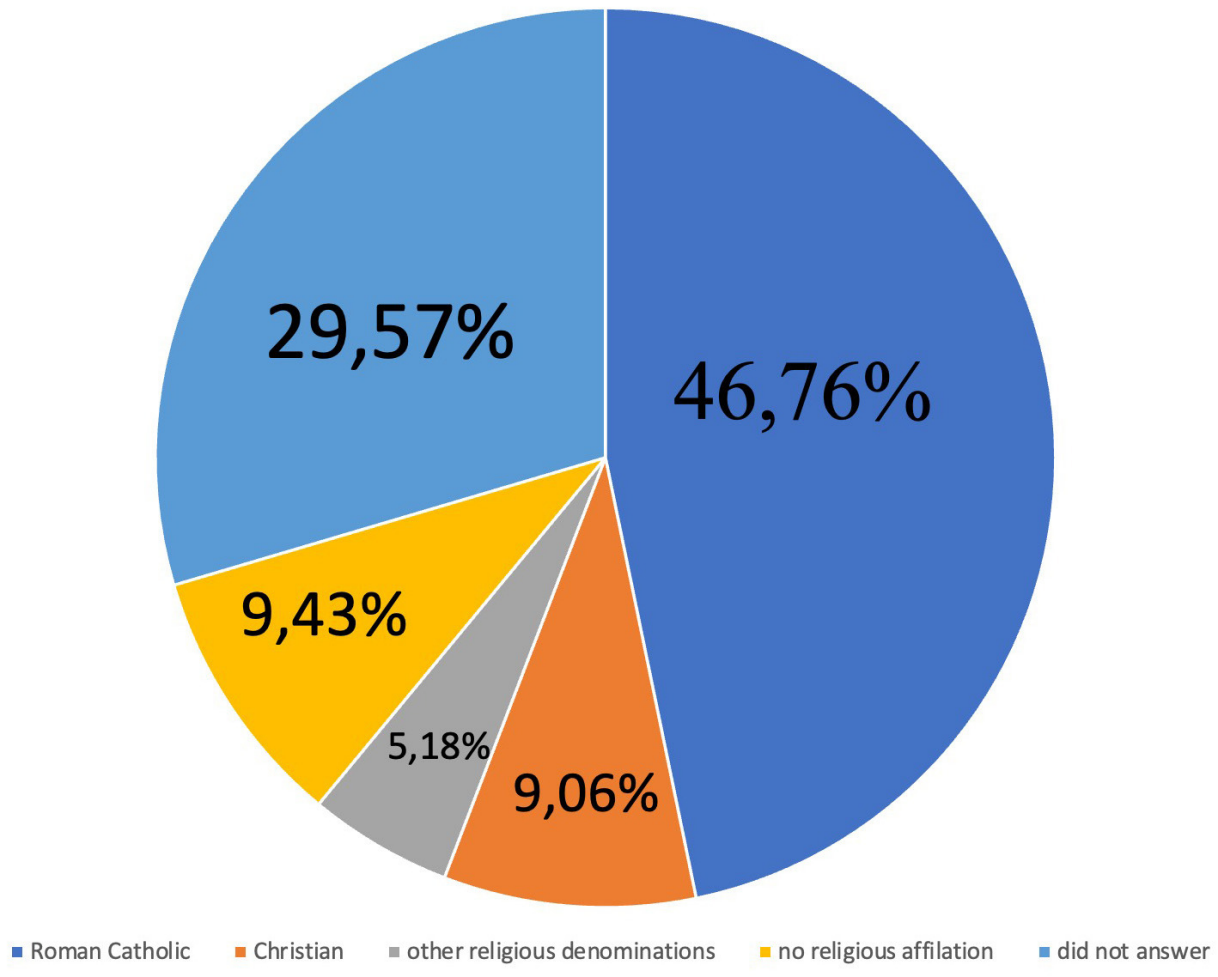
Percentage of all respondents.

The research included 541 people of whom 35.12% were male (*n* = 190) and 64.51% (*n* = 349) were female, 0.37% (*n* = 2) did not specify gender. There were 246 in the first study group and 295 respondents in the second (Table 1). The number of respondents declaring Roman Catholic faith was *n* = 253 (46.76%), the number of Christians belonging to other Christian rites was *n* = 49 (9.06%), affiliation with other religious denominations was *n* = 28 (5.18%), no religious affiliation was declared by *n* = 51(9.43%) respondents, those who did not answer the question about their faith were *n* = 160 (29.57%; Figure 1).

**Table 1:** Distribution of religious affiliation among pandemic and post-pandemic groups.

**Demographic variable**	**Pandemic group (*n* = 246)**	**Post-pandemic group (*n* = 295)**	**Total (*n* = 541)**
Roman Catholic	115 (46.7%)	138 (46.8%)	253 (46.76%)
Other Christian	24 (9.8%)	25 (8.5%)	49 (9.06%)
Other Religions	14 (5.7%)	14 (4.8%)	28 (5.18%)
No Religious Affiliation	28 (11.4%)	23 (7.8%)	51 (9.43%)
Unanswered	65 (26.4%)	95 (32.2%)	160 (29.57%)

Comparing the answers of all respondents Roman Catholics rated the accessibility of healthcare services prior to the COVID-19 pandemic higher than respondents declaring no religious affiliation. There were no statistically significant differences in perceptions of the accessibility of healthcare services both before (*p*_1_) and during (*p*_2_) the COVID-19 pandemic in relation to declared religious affiliation among all respondents (*p*_1_ = 0.377; *p*_2_ = 0.304_)_ as well as in the first (*p*_1_ = 0.203; *p*_2_ = 0.211) and second (*p*_1_ = 0.505; *p*_2_ = 0.144_)_ study groups. Above that, no statistically significant differences were shown in the utilization of healthcare services with regard to religious affiliation among all respondents (*p* = 0.535) as well as in the first (*p* = 0.969) and second (*p* = 0.456) study groups.

In the research group examined during the third wave of the COVID-19 pandemic (*n* = 246), ratings of perceptions of accessibility to healthcare services were higher among respondents for whom religion is important in life. In the post-pandemic study group (*n* = 295), perceptions of healthcare accessibility did not differ based on the significance of religion in respondents' lives

There were no differences among all respondents, as well as between the first and second groups, in assessing perceptions of the accessibility of healthcare services both before and during the COVID-19 pandemic.

The analysis of gender distribution across religious affiliations did not reveal a statistically significant association between these two variables (*p* = 0.156). The results suggest that religious affiliation does not significantly differ by gender in this study population.

## Discussion

In Poland, the most numerous religious community is the Roman Catholic Church. While much of the analysis has focused on Roman Catholics and the religiously unaffiliated, the study also included individuals from other Christian denominations and non-Christian religious traditions, though these groups constituted a relatively small percentage of the study population. For analytical purposes, Protestants and Orthodox Christians were categorized under “other Christian denominations,” while individuals identifying with non-Christian faiths were grouped under “other religions.” Differences in healthcare perceptions among these groups can be attributed to varying theological views, community support networks, and historical relationships with healthcare systems.

Protestant in Poland, although a minority, exhibited higher levels of trust in private healthcare services compared to public institutions. This may be linked to a historical emphasis on self-reliance and community-based health initiatives observed in Protestant communities (13). Additionally, Protestant groups tend to have stronger individualistic perspectives on health, which may influence attitudes toward healthcare accessibility and utilization (29). Orthodox Christian, primarily from ethnic minority groups, often faced language barriers and cultural adaptation issues in Poland's predominantly Roman Catholic healthcare system. Research suggests that Orthodox believers may prioritize spiritual healing and clergy consultations alongside medical treatment, which could explain their unique perceptions of healthcare access (12).

When it comes to other religious groups (Judaism, Islam, Hinduism, etc.) Research shows that individuals' belonging to non-Christian religious traditions reported higher perceived barriers to healthcare due to cultural and dietary restrictions, language barriers, and lower representation of religiously sensitive healthcare providers (30). For example, studies on Muslim patients highlight the importance of gender-concordant healthcare providers and halal dietary accommodations, which are not always adequately provided in Polish hospitals (27). Similarly, Jewish people emphasized the need for Sabbath-compliant healthcare access, which may influence their perception of medical availability (31).

Quite a few people in the study did not answer the question about their faith. Religion is considered to be a personal and intimate matter. Many people may feel that such questions invade their privacy. Some may avoid answering the question about religion because they do not want their religious beliefs to be categorized in any way. Such respondents may prefer to keep their views out of the questionnaire, especially if they feel that religion does not play an important role in their lives (32, 33)^1^.

Roman Catholic respondents' rated the accessibility of healthcare services during the COVID-19 pandemic higher than those of respondents with no religious affiliation. This may be because Roman Catholic respondents were able to benefit from the strong support of their religious communities, which played an important role during the pandemic. Parishes and church organizations were often involved in outreach activities, such as organizing transport to hospitals, helping people access medical care, or providing emotional and/or spiritual support (34, 35). In addition, religious people may have a greater sense of optimism and hope, which may have influenced the assessment of their situation, including the accessibility of healthcare. They may also have had stronger social bonds that were crucial during the pandemic. Support from family, friends, the parish community or charities may have made Roman Catholic respondents feel more able to rely on help in accessing healthcare services or getting the information they needed. Respondents with no religious affiliation may have had weaker social support structures, which may have influenced their less positive perceptions of healthcare accessibility (12, 36, 37).

Although Roman Catholic respondents rated accessibility to health services higher during the COVID-19 pandemic, the study found that religion did not affect perceived accessibility to health services before or during the COVID-19 pandemic, nor did it affect the utilization of healthcare services. Poland has a largely public medical system, with healthcare services available to all citizens regardless of their religion. Religiosity as a factor may influence personal feelings of security or support, but does not necessarily change actual access to health services. Respondents may not perceive differences in access because the system is standardized, which may have negated the impact of religion as a differentiating factor (18, 38). Faith-based organizations contributed significantly to the dissemination of public health messages during the pandemic, fostering trust in medical recommendations (39). As a follow up, while religion can provide emotional support, and religious communities such as the Catholic Church offered help during the pandemic, formal access to health services (e.g., doctor's appointments, hospitalization) depends mainly on systemic factors such as health policy, infrastructure and hospital resources (31, 40, 41). In addition, during the pandemic, numerous state-level measures were introduced to increase access to healthcare, e.g. telemedicine, increased healthcare resources. These nationwide measures may have minimized differences in perceived access, regardless of religious affiliation. In the face of a public health threat such as a pandemic, people's priorities changed and health issues began to dominate over religious ones. People were able to focus on the need to secure access to health care for themselves and their loved ones, which may have reduced the influence of religious affiliation on decisions to use health services (42, 43).

In the study carried out, those in the group surveyed during the third wave of the pandemic who reported that religion was important in their lives rated their perceived access to healthcare higher than those surveyed in the post-pandemic period. In the face of a health disruption such as the COVID-19 pandemic, many people may begin to see religion as an important emotional and spiritual support. People for whom religion is important may find a sense of hope and support in prayer and religious practices, which is reflected in their positive perceptions of access to healthcare services. Support from religious communities may increase feelings of safety and belonging, which may influence perceived accessibility of health services. In the third wave of the pandemic, many people experienced intense fear and uncertainty about COVID-19, which may have led to greater interest in utilizing healthcare services. Moreover, religious people may have believe that their religiosity would help them overcome difficulties. In contrast, once the pandemic was over, people may have a different attitude toward utilizing healthcare services, which may affect their perceptions. This include feeling that health problems are no longer as urgent, or becoming discouraged about the health system as a result of negative experiences during the pandemic (43, 44).

Social amnesia may also be one of the reasons for these results (45). The post-pandemic COVID-19 community forgetfulness syndrome is a phenomenon that affects many communities around the world. After an intense period when the pandemic dominated the media and daily life, the population may tend to quickly forget the difficulties and challenges of the healthcare sector during COVID-19 (46). The post-pandemic COVID-19 social amnesia is a phenomenon that requires detailed understanding and analysis in the context of the impact of weaker healthcare during the disruption. The pandemic caused by the SARS-CoV-2 virus caused a global health challenge that focused the attention of health systems on providing an immediate response to the growing demand for COVID-19-related treatment. During this time, regular medical care for patients with other chronic diseases and health needs was reduced, which could lead to delays in diagnoses, interruptions in therapies and an overall deterioration in population health (47).

The results align with previous studies indicating that religious affiliation provides emotional and social support, enhancing perceptions of healthcare access during crises (12, 13). However, the lack of significant differences in actual healthcare access highlights the dominance of systemic factors over individual religious beliefs (22, 48). During the pandemic, religious communities played a supportive role, organizing transport and offering mental health resources (31, 34, 49). Yet, formal healthcare access remained dictated by government policies and institutional capacity rather than religion. These findings suggest that while religious belief influences individual perception, it does not alter tangible healthcare accessibility (50). A notable trend was the decline in perceived accessibility post-pandemic, potentially attributed to a “forgetfulness syndrome,” where individuals became less engaged with healthcare as the disruption subsided (43). This study aimed to determine the impact of the religious component on the perception of access to healthcare services during the COVID-19 pandemic and the post-pandemic period in Poland. Future research should explore longitudinal trends in religion's impact on healthcare perceptions and access.

## Conclusion

The study explores the relationship between religious affiliation and perceptions of healthcare access in Poland during and after the COVID-19 pandemic. Findings suggest that while religion plays a role in shaping personal attitudes toward healthcare, the broader accessibility of healthcare services is influenced by multiple factors beyond religious identity. The study acknowledges the limitations of comparing a dominant religious group with smaller religious minorities, given the disproportionate representation of these groups in the sample. While the influence of minority religious affiliations may not be as statistically significant in shaping overall findings, their inclusion remains relevant for understanding the diversity of perspectives on healthcare access. Future research should consider more targeted sampling strategies to enhance the representation of minority religious groups, ensuring a more balanced and comprehensive analysis of religious influences on healthcare perceptions.

## Data Availability

The raw data supporting the conclusions of this article will be made available by the authors, without undue reservation.

## Footnotes

^1^ https://stat.gov.pl/files/gfx/portalinformacyjny/pl/defaultaktualnosci/5500/8/1/1/zycie_religijne_w_polsce_wyniki_badania_spojnosci_spolecznej_2018.pdf
